# Primary results of the Spanish Cryoballoon Ablation Registry: acute and long-term outcomes of the RECABA study

**DOI:** 10.1038/s41598-021-96655-3

**Published:** 2021-08-26

**Authors:** Ángel Ferrero-De-Loma-Osorio, Rocío Cózar, Arcadio García-Alberola, Ermengol Valles, Alberto Barrera, Jorge Toquero, Jose Miguel Ormaetxe, Juan Martínez Sánchez, Ricardo Ruiz-Granell, Pablo Bastos Amador, Jose Manuel Rubio, Julio Martí-Amor, Patricia Pascual, Irene Molina, Jesús Daniel Martínez-Alday, RECABA Investigators

**Affiliations:** 1 Arrhythmia Unit, Cardiology Department, Hospital Clínico Universitario, INCLIVA Foundation, Avenida Blasco Ibáñez 17, 46010 Valencia, Spain; 2 Arrhythmia Unit, Cardiology Department, Hospital Universitario Virgen Macarena, Sevilla, Spain; 3 Arrhythmia Unit, Cardiology Department, Hospital Universitario Virgen de la Arrixaca-IMIB, Murcia, Spain; 4 Arrhythmia Unit, Cardiology Department, Hospital del Mar, Barcelona, Spain; 5 Arrhythmia Unit, Cardiology Department Hospital Virgen de la Victoria Málaga, Málaga, Spain; 6 Arrhythmia Unit, Cardiology Department, Hospital Universitario Puerta de Hierro, Madrid, Spain; 7 Arrhythmia Unit, Cardiology Department, Hospital de Basurto, Bilbao, Spain; 8 Arrhythmia Unit, Cardiology Department, Hospital Universitario Fundación Jiménez Díaz, Madrid, Spain; 9 Medtronic Iberia, S.A., Madrid, Spain

**Keywords:** Cardiology, Atrial fibrillation

## Abstract

Cryoablation is safe and effective for the treatment of atrial fibrillation (AF) in controlled clinical trials, but contemporary real-world usage and outcomes are limited. The Report of the Spanish Cryoballoon Ablation Registry (RECABA) was designed to evaluate acute and 12-month outcomes of cryoballoon ablation for the treatment of AF in Spain. Patients from 27 Spanish centers were prospectively enrolled. Patients were treated with cryoballoon ablation and managed according to standard of care protocols at each center. The primary endpoint was ≥ 30 s freedom from AF at 12-month after a 3-month blanking period. Secondary endpoints included a description of patient characteristics, cryoablation procedural strategy and safety, and predictors of efficacy. In total, 1742 patients (71.4% PAF, 68.8% male, mean age 58.02 ± 10.40 years, 76.1% overweight or obese, CHA_2_DS_2_-VASc index 1.40 ± 1.28) were enrolled. Patients received 7.2 ± 2.67 cryo-applications. PV potentials could be detected in 61% of the PVs during ablation, with a mean time to block of 52.9 ± 37.02 s. Acute PVI was observed in 97% of PVs with 75.8% isolated with the first cryo-application. Mean procedural time was 113 ± 41 min. Acute complications occurred in 4.4% of the cases. With follow-up in 1628 patients, AF-free survival was 78.5% (PAF: 80.6% vs PersAF 73.3%; p < 0.001). Left atrium enlargement, female sex, non-PAF, and early recurrence were independent predictors of AF recurrence (p < 0.05). RECABA provides detailed insight into current dosing practices and demonstrates cryoablation is safe and effective in real-world use.

**ClinicalTrials.gov number**: NCT02785991.

## Introduction

Cryoballoon ablation (CBA) is a well-established technique for the treatment of atrial fibrillation (AF), that has been demonstrated to achieve similar safety and efficacy as radiofrequency ablation (RFA) with shorter procedure times^1^. Previous studies have suggested a benefit to intervention with ablation before drug failure, because a shorter “diagnosis-to-ablation” time is associated with better outcomes^2,3^.

While the safety and efficacy of AF ablation has been established, it was only until recently that outcomes of AF ablation were examined within large, prospectively enrolled cohorts of patients with AF. The recent report from the *Cryo AF Global Registry* described primary outcomes of cryoablation used according to local practice around the world^4^. Country specific evaluations such as the GWTG-AFIB study, the FREEZE Cohort, and the 1STOP registry describe outcomes of cryoablation in the USA, Germany, and Italy, which have had many years of experience with the cryoablation catheter^5–7^. Uniquely, a recent report from Miyazaki et al. described the first safety experience with cryoablation in Japan^8^. A comprehensive examination of modern cryoablation outcomes in an early, pan-country experience with cryoballoon ablation has yet to be reported.

The Spanish Catheter Ablation Registry is published annually as the official report of the Spanish Society of Cardiology Working Group on Electrophysiology and reflects general activity in Spanish electrophysiology units but does not describe AF ablation procedures in detail; therefore, patient selection, procedural techniques, and outcomes in real-world use in Spain have not been reported^9^. The *Spanish Registry of Cryoballoon Ablation* (RECABA) prospective multicenter observational study aimed to comprehensively evaluate daily clinical practice and the current state of cryoballoon procedures in Spain.

## Methods

### Study design and population

**Figure 1:**
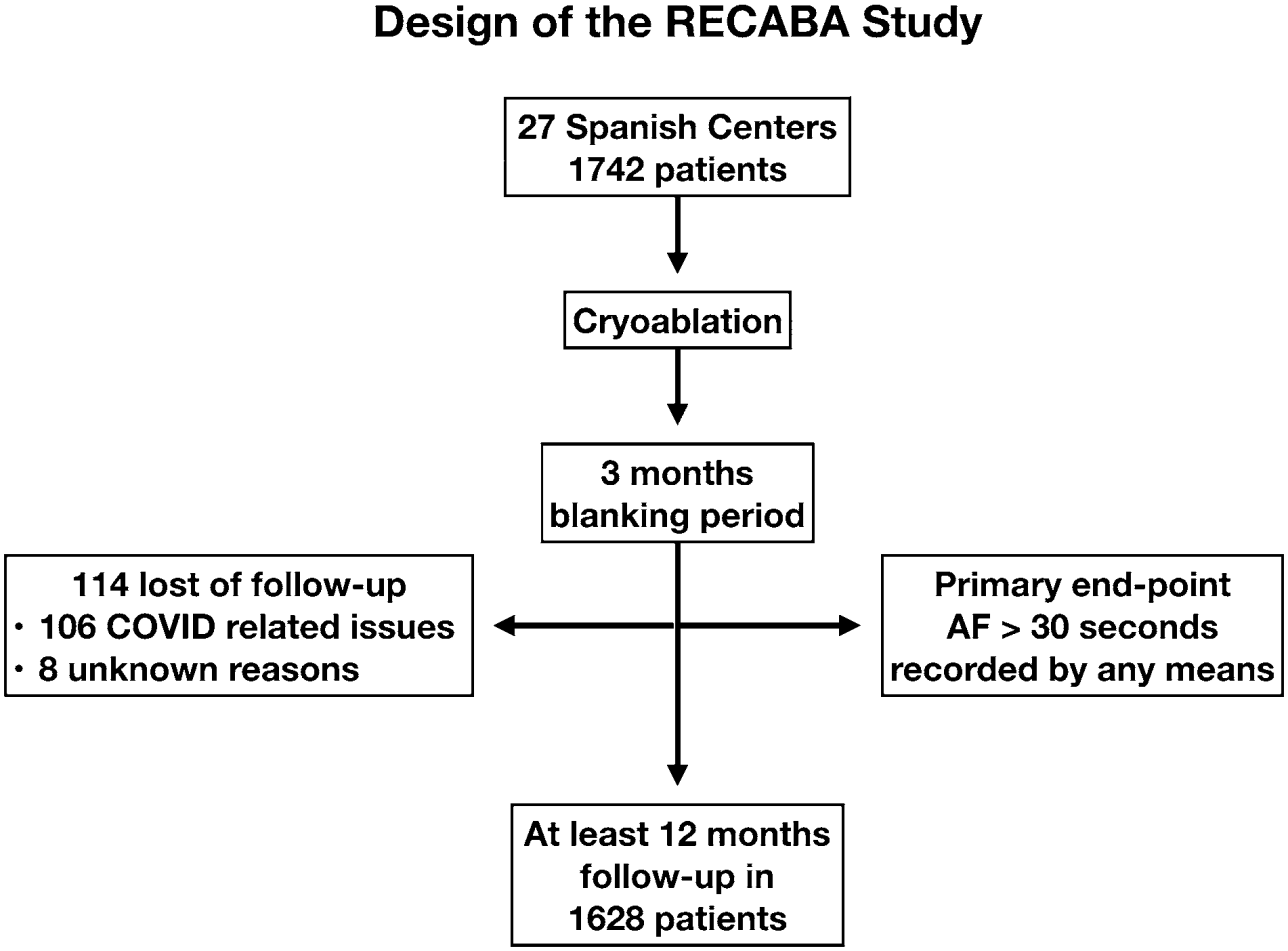
Visual representation of RECABA protocol.

The Registro Español de CrioAblación con BAlón (RECABA; NCT02785991) is an observational, prospective, national, multicenter (Supplementary Material Online S1, Table S1) study of patients undergoing CBA for AF in Spanish centers with experience in the technique (at least ten procedures per year). Patients were enrolled between September 2016 and January 2019. For inclusion in the study, patients were required to (1) be older than 18 years of age, (2) be eligible for CBA for AF, (3) have a life expectancy greater than 1 year, and (4) sign the informed consent. Patients with both first and repeat procedures were enrolled. AF was classified as paroxysmal (PAF) or persistent atrial fibrillation (PerAF) according to the current ESC guidelines^10^. Clinical data were collected at the baseline procedure and at the annual follow-up by the coordinator responsible for each center through an electronic data collection system for clinical trials (eCRF). Data collection, management and quality control are detailed in Supplementary Material Online (S2). Figure 1 shows a visual representation of the protocol and its timeline.

Ethics Committee approval was obtained according to local legislation. The study was conducted in compliance with the most recent version of the Declaration of Helsinki, Spanish laws and regulations (Royal Decree 1090/2015, Royal Decree 1616/2009, Order SAS/3470/2009 of 16 December). This observational study did not require authorization by the Spanish Agency of Medicines and Medical Devices (AEMPS), as stipulated in Royal Decrees 1090/2015 and 1616/2009, since it is a clinical investigation with CE marked medical devices used in accordance with the clinical purpose of the device. The study was assessed and approved by the IRB, Comité Ético de Investigación Clínica de Euskadi (CEIC-E) on May 9, 2016, and by the Ethical Committee of Hospital de Mar, Comité Ético de Investigación Clínica del Consorci Mar Parc de Salut de Barcelona (CEIC-Parc De Salut Mar) on June 21, 2016. All patients signed informed consent before inclusion in the registry.

### Objective and endpoints

The main objective of the RECABA was to report standard clinical practice for PVI using CBA in Spanish hospitals. Standard clinical practice was evaluated by assessing patient characteristics, procedural and CBA techniques, procedure-related complications, the long-term follow-up strategy and efficacy across Spanish centers. The primary endpoint of the study was AF free survival at the 12-month follow-up. Secondary endpoints included the following: (1) a description of baseline characteristics of subjects who underwent CBA, (2) the acute efficacy, safety (the rate of all procedure-related adverse events), and efficiency of the procedure (e.g. electrical PVI, PV potential monitoring, cryoapplications analysis, related complications, and procedural related times), (3) description of the dosing strategies utilized, and (4) identification of the factors that predicted AF recurrence during follow-up.

### Cryoballoon ablation procedure

General pre-ablation management and the CBA procedure were done in accordance with each center’s standard protocol for pre-ablation examination, anesthesia and sedation, auxiliary technology for transseptal puncture, anatomical assessment, and phrenic nerve monitoring during right-sided ablation. Nevertheless, the CBA procedure was similar in all hospitals as cryoballoon-over-the-wire techniques were required to follow the internationally accepted techniques described in detail in recent publications and in the Supplementary Material Online (S3)^11,12^. Of note, the following cryo-dosing parameters followed the usual practice of each hospital including duration of cryoapplications, post-ablation waiting time, and post ablation acute PVI adenosine testing. Initially, an extra application of cryothermia (bonus application) was carried out after the application that had blocked the pulmonary vein. In recent years, after the publication of studies showing that this extra application is not necessary, the tendency is to avoid it, since it does not provide clinical benefit and could be related to an increase in complications^13^. In this Registry, the bonus/non bonus freeze strategy also followed the usual practice of each hospital.

### Post-ablation management and follow-up

Oral anticoagulation (OAC) and anti-arrhythmic drug (AAD) management were done in accordance with each center’s standard protocol and at the discretion of the cardiologist. Patients could continue taking AAD in the absence of recurrences. Patients were followed with outpatient visits using 24 h to 30-day Holter monitoring systems over the 12-month follow-up. Arrhythmia recurrence could also be monitored via electrograms registered in patients with an implanted device prior to (but not after) enrollment in the study. Patients were also instructed to perform an ECG when they present with symptoms. AF recurrence was defined as any AF episode that lasted longer than 30-s accordingly documented (on ECG, Holter monitor, event recording systems or implantable devices). A 3-month blanking period was established, during which detected AF episodes were excluded from the primary endpoint.

### Statistical analysis

Quantitative variables are expressed as mean, median, standard deviation and interquartile range as appropriate. Differences in quantitative variables were evaluated through the student *t*-test for independent samples (or analysis of variance depending on the number of groups compared) and between paired variables through the student *t-*test for related samples or the analysis of variance for repeated measures. Categorical data were compared with the chi-square test or Fisher’s exact test, depending on the number of categories. The probability of recurrence was predicted using a logistic regression, and a survival analysis predicted the most likely time of recurrence. Univariate models considering each potential predictor were estimated. Subsequently, a multivariate model considering predictors from the baseline visit and procedural characteristics were estimated. An overall multivariate model including significant variables was estimated with non-significant variables as covariates. Time to recurrence was studied using Kaplan–Meier estimates and categorical predictive covariates were included as grouping variables and compared using the log-rank statistic. The Cox-proportional hazard regression method was used to estimate the conditional hazard rate. In order to study AAD use retention at follow-up visits, a logistic loglinear model was estimated, including AF type, AAD use after cryoablation procedure and AF recurrence. Effect confidence intervals and model standardized residuals were obtained. Chi-square for independence was used as test statistic for independence. All analyses were carried out using IBM SPSS v26.0 software. A nominal 5% significance level was assumed in all analysis and adjustments for multiple comparisons were completed when needed.

## Results

### Study population

A total of 1742 patients eligible for CBA for the treatment of paroxysmal (1238; 71.1%) or persistent (504; 28.9%) AF were prospectively included from 27 Spanish Centers. The mean number of patients included per center was 64.5 ± 53.21, (13 centers enrolled less than 50 patients, 9 enrolled between 51 and 100, 3 between 101 and 150 and 2 enrolled more than 150 patients). All but one enrolled patient (who opted out of the procedure after singing informed consent) underwent a CBA. Of all procedures, 1665 (96.6%) were first CBA procedure and 77 (4.4%) were repeat ablation procedures.

Clinical characteristics of the study population are shown in Table 1. Most patients were male (68.8% versus 31.2%; p < 0.001), under 65 years old (70.7%), and were overweight or obese (76.1%). Only 52 patients (3%) were older than 75 years. One patient was younger than 20 years and 4 patients were older than 80 years of age. The most frequently observed cardiovascular risk factors were hypertension (46.3%) and dyslipidemia (34.7%). The population presented a low embolic risk overall (mean CHA_2_DS_2_-VASc index 1.40 ± 1.28), but CHA_2_DS_2_-VASc was ≥ 2 in 39.8% of the cohort. Only 18.9% of patients had structural heart disease (most frequently tachycardia-related cardiomyopathy and coronary artery disease) and left ventricular ejection fraction was preserved in most patients (≥ 50% in nearly 90% of the patients). LA was enlarged (LA > 40 mm or area > 20 cm^2^) in over half of the patients (most frequently mild). Oral anticoagulation (most frequently direct anticoagulation drugs) and AADs were taken at baseline in 75.5% of patients. In 148 patients (8.5%) CBA was used as a first-line treatment. Patients had PAF with a mean time since diagnosis of more than 1 year in 85.6% of cases.

**Table 1:** Clinical baseline characteristics.

Baseline characteristics	n = 1742
**Age (years) (mean ± SD (range))**	58.02 ± 10.40 (20–85)
≤ 35 years old; (n, %)	49 (2.8)
36–64 years old; (n, %)	1183(67.9)
65–74 years old; (n, %)	458 (26.3)
≥ 75 years old; (n, %)	52 (3)
Gender, male; n (%)	1199 (68.8%)
**BMI (kg/m**^**2**^**)**	28.1 ± 4.26
BMI > 25–30 kg/m^2^ (overweight); (n, %)	814 (46.7)
BMI > 30 kg/m^2^ (obesity); (n, %)	512 (29.4)
**AF type; n (%)**	
Paroxysmal	1237 (71.1%)
Persistent	504 (28.9)
**Prior catheter ablation; n (%)**	77 (4.4)
Previous cryoballoon ablation	33 (42.9)
Previous radiofrequency ablation	44 (57.1)
Time since diagnosis < 1 year; n (%)	245 (14.4)
**Cardiovascular risk factors; n (%)**	
Hypertension	806 (46.3)
Diabetes mellitus	158 (9.1)
Smokers	224 (13.3)
Dyslipidemia	605 (34.7)
Ischemic stroke/TIA	93 (5.3)
Vascular disease	97 (5.6)
**CHA**_**2**_**DS**_**2**_**-VASc; n (%)**	
0	494 (28.7)
1	543 (31.5)
2	346 (20.1)
3	221 (12.8)
4	81 (4.7)
5	31 (1.8)
> 5	6 (0.4)
High level of exercise* n (%)	80 (4.8)
**Sleep apnea; n (%)**	206 (12.9)
CPAP AHS treated patients; n (%)	141 (68.4)
**Heart disease; n (%)**	329 (18.9)
Coronary artery disease	112 (34.0)
Idiopathic dilated cardiomyopathy	40 (12.2)
Hypertrophic cardiomyopathy	26 (7.9)
Valvular heart disease	29 (8.8)
Tachycardia-related cardiomyopathy	113 (34.3)
Other cardiac disease	24 (7.3)
**LVEF; n (%)**	
≤ 35%	67 (4.1)
36–50%	97 (6.0)
> 50%	1456 (89.9)
**Left atrium dilatation; n (%)**	825 (51.4)
Mild (area 20–30 cm^2^)	384 (64.9)
Moderate (area 31–40 cm^2^)	157 (26.5)
Severe (area > 40 cm^2^)	51 (8.6)
Left ventricular hypertrophy; n (%)	255 (15.8)
Congestive heart failure; n (%)	141 (8.1)
**Other associated arrhythmias; n (%)**	383 (22.1)
Atrial tachycardia	19 (1.1)
Common atrial flutter	298 (17.1)
Atypical atrial flutter	26 (1.5)
Other arrhythmias	40 (2.3)
Implanted cardiac pacemaker; n (%)	56 (3.2)
**AAD treatment**	
Prior AAD per patient (mean ± SD)	1.27 ± 0.72
Previously AAD failed; n (%)	
0	148 (8.7)
1	1068 (62.6)
2	394 (23.1)
> 2	97 (5.6)
**Patients taking AAD; n (%)**	1307 (75.3)
Flecainide	781(44.8)
Amiodarone	400 (23.0)
Propafenone	57 (3.3)
Sotalol	36 (2,1)
Other AAD	90 (5.2)
Patients taking betablockers; n (%)	1161 (67.0)
Patients taking oral anticoagulation; n (%)	1309 (75.5)
**Direct anticoagulation agents**	915 (69.9)
Rivaroxaban	316 (34.5)
Apixaban	303 (33.1)
Dabigatran	186 (20.3)
Edoxaban	111 (12.1)
Left common PV	277 (16.0)
2 left PV	1443 (83.6)
> 2 left PV	6 (0.3)
Right common PV	30 (1.7)
2 right PV	1569 (91.1)
> 2 right PV	123 (7.1)

Percentage values have been calculated for valid responses and on the total of the sample. Quantitative data are the mean ± standard deviation.

*BMI* body mass index, *AF* atrial fibrillation, *TIA* transient ischemic attack, *CHA*_*2*_*DS*_*2*_* VASc*_*2*_ Score for AF and stroke risk, *AHS* apnea–hypopnea syndrome, *LVEF* left ventricular ejection fraction, *AAD* antiarrhythmic drugs, *PV* pulmonary veins, *LCT* left common trunk, *RCT* right common trunk.

*High level of exercise defined as > 300 min/week.

### Procedural characteristics

**Figure 2:**
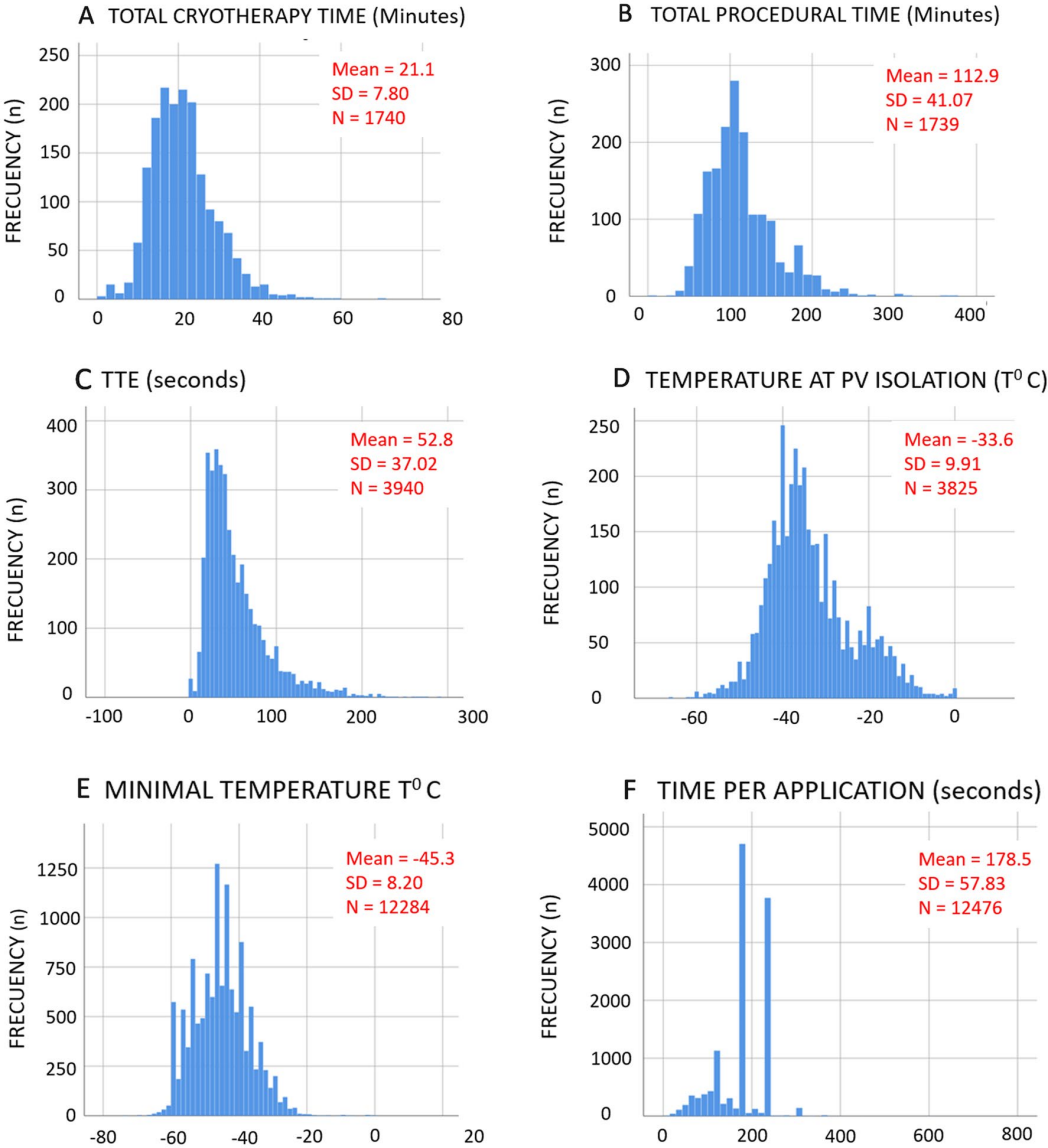
Histograms of the distribution of frequencies: total cryotherapy time (**A**), total procedural time (**B**), time-to-effect (**C**), temperature at PVI (**D**), minimal temperature during application (**E**), time per-application (**F**). *SD* standard deviation.

Procedure characteristics were analyzed from a total of 1741 CBA procedures (Table 2). In nearly 65% of patients pre-procedural imaging was performed to assess PV anatomy. Most patients (1316 patients; 76.5%) had 4 independent PVs. Only 15% of procedures were performed under general anesthesia. The 28 mm Arctic Front Advance cryo-balloon was used in 93.8% of the cases. A bonus freeze strategy was systematically employed in 553 procedures (33.7%) and was never employed in 679 (41.4%) procedures. In 407 procedures (24.8%), a bonus application was administered depending on the perception of the quality of the previous application by the operator. Procedure times (cryotherapy, left atrium, total procedure, and X-ray exposure times) are shown in Table 3. Mean number of applications and mean total cryotherapy time was 7.2 ± 2.67 and 21.1 ± 7.80 min per patient, respectively. Patients treated with a no-bonus strategy received significantly fewer (but longer duration) applications, had a shorter time to effect (TTE), and received significantly shorter cryotherapy, LA and fluoroscopy exposure times (p < 0.001); however, total procedure time did not reach statistical significance between no-bonus and bonus strategies (p = 0.07). Figure 2 shows the distribution of total cryotherapy (A) and procedural times (B).

**Table 2:** Procedural characteristics.

Characteristic	n = 1741
Pre-procedural imaging; n (%)	1122 (64.4)
Computerized tomography	938 (84.0)
Magnetic resonance imaging	176 (15.8)
Intra-procedural imaging; n (%)	260 (14.9%)
**Rhythm at time of ablation; n (%)**	
Sinus rhythm	1275 (73.9)
Atrial fibrillation	424 (24.6)
Common atrial flutter	33 (1.9)
Atypical atrial flutter	7 (0.4%)
**Anesthesia and sedation; n (%)**	
General anesthesia	261 (15.0)
Deep sedation	537 (36.0)
Superficial sedation	953 (64.0)
Invasive control of blood pressure; n (%)	710 (41.1)
**Diagnostic catheters (Achieve catheter included); n (%)**	
1	298 (17.2)
2	811 (46.9)
> 2	621 (35.9)
Arctic front advance 28 mm; n (%)	1615 (93.8)
Imaging during transeptal approach; n (%)	256 (14,7)
Transesophageal echocardiography	149 (58.2)
Intracardiac echocardiography	107 (41.8)
**Phrenic nervous monitoring technique; n (%)**	
Palpation	1716 (99.1)
Compound motor action potential monitoring	370 (21.4)
X-ray real time visualization	388 (22.4)
Bonus cryodosing strategy; n (%)	553 (33.7)
Total cryoapplication duration (min)	21.1 ± 7.80
Left atrium time (min)	74.6 ± 27.89
Waiting time post ablation; n (%)	473 (28.5)
Waiting time post ablation (min)	17.2 ± 9.55
Adenosine test; n (%)	71 (4.2)
Cardioversion during ablation; n (%)	542 (31.6)
Cavotricuspid isthmus ablation n (%)	131 (7.5)
Protamine administration per protocol; n (%)	877 (51.3)
Subcutaneous "Z"-stitch; n (%)	1244 (72.3)
Time to discharge (days)	1.2 ± 2.01

Percentage values have been calculated for valid responses and on the total of the sample. Quantitative data are the mean ± standard deviation.

**Table 3:** Procedural characteristics by cryoablation strategy.

Characteristic	Overall	Bonus strategy	Non-bonus strategy	p-value
Applications per patient	7.2 ± 2.67	8.6 ± 2.51	6.5 ± 2.49	< 0.001
Cryotherapy time per application (s)	178.5 ± 57.83	173.8 ± 55.25	181.5 ± 59.17	< 0.001
Total cryoapplication duration (min)	21.1 ± 7.81	24.4 ± 8.04	19.6 ± 7.21	< 0.001
Left atrium time (min)	74.6 ± 27.89	80.7 ± 27.69	70.9 ± 27.38	< 0.001
Total procedure time (min)	113.0 ± 41.08	115.6 ± 42.12	111.8 ± 40.55	0.072
Total fluoroscopy time (min)	23.9 ± 1.6	25.0 ± 15.06	21.1 ± 17.55	< 0.001
Intraprocedural complications; (n, %)	77 (4.4)	32 (5.7)	45 (3.7)	0.085

Quantitative data are expressed as (mean ± SD).

### Acute procedural outcomes and applications analysis

A total of 12,495 cryoapplications applied to 6715 PVs (277 left and 30 right common trunks) were performed, for a mean of 1.8 applications per vein. At the end of the procedure 97% of PV were isolated, of which 75.8% were isolated with the first cryoapplication. In 13% of the cases patients received only one application per vein for PVI (4 applications per intervention). The rest of the patients (86.2%) received more than one application in any of the locations. Global and per vein data analysis are summarized in Table 4. Potentials inside the PV could be monitored during ablation in 61% of PVs with a mean TTE of 52.9 ± 37.02 s. Figure 2 shows the histograms of the distribution of TTE (C), temperature at PVI (D) and the minimal temperature reached (E). The most frequent cryoapplications duration was 180 or 240 s (F).

**Table 4:** Acute procedural results. Global and per vein application analysis.

Overall				
Total veins (n)				6715
Total applications				12,491
Applications per vein (n)				1.8 ± 1.11
Time per application (s)				178.5 ± 57.83
PV potentials; n (%)				4098 (61.0)
TTE (s)				52.9 ± 37.02
Temperature at PVI (°C)				− 33.6 ± 9.92
PVI with 1st application; (n/%)				75.8 (6381)
Minimum temperature (°C)				− 45.4 ± 8.20
Rewarming time (s)				38.7 ± 20.15

Per vein analysis	LSPV ^A^	LIPV^B^	RSPV^C^	RIPV^D^
Total veins (n)	1537	1534	1691	1660
Total applications (n)	2828	2766	3059	3148
Applications per vein	1.8 ± 1.07	1.8 ± 1.01	1.8 ± 1.06	1.9 ± 1.17
Time per application (s)	179.7 ± 56.14^BD^	179.7 ± 59.74^AD^	173.6 ± 58.76*	179.4 ± 57.65^AB^
PV potentials (%)	1110 (72.4)*	967 (63.2) *	997 (59.2) *	834 (50.6) *
TTE (s)	52.1 ± 34.14^BC^	51 ± 34.3^AC^	49.0 ± 38.88^AB^	59.0 ± 39.62*
Temperature at PVI (°C)	− 35.2 ± 9.29^D^	− 31.3 ± 9.7*	− 33.2 ± 10.78^D^	− 34.4 ± 9.43^C^
PVI with 1st application; n (%)	1487 (77.67)^BCD^	1455 (78.35)^AC^	1610 (75.0)^ABD^	1552 (73.7)^AD^
Total cryotherapy time (s)	330.3 ± 173.92^BCD^	323.8 ± 162.16^AC^	314.0 ± 174.86^AB^	340.4 ± 193.07^A^
Minimum temperature (°C)	− 46.4 ± 8.04*	− 43.4 ± 7.84*	− 47.3 ± 8.07*	− 44.4 ± 8.20*
Rewarming time (s)	43.3 ± 22.82*	36.7 ± 18.94^D^	41.0 ± 19.63*	34.3 ± 17.80^B^

Differences on averages by patient-location, since multiple applications could have received at each patient-location (on average 1.8 applications over each location) are shown, using a mixed-effects model and adjusting for multiple comparisons (Bonferroni). Locations with a significantly different value (p < 0.001) differing from all other locations are flagged with an asterisk. When the average compared is not different from another location (p > 0.05), the value is labeled with a letter corresponding to the non-significant locations (labeled from left to right: A, B, C, D).

Quantitative data are the mean ± standard deviation.

*PV* pulmonary vein, *PVI* PV isolation, *TTE* time to effect, *LSPV* left superior pulmonary vein, *LIPV* left inferior pulmonary vein, *RSPV* right superior pulmonary vein, *RIPV* right inferior pulmonary vein.

### Periprocedural-related complications

Periprocedural and procedural-related complications occurred in 120 patients (6.9%). Acute intraprocedural complications were reported in 77 patients (4.4%). The most frequent adverse event observed was phrenic nerve injury (49 patients; 3%), of which 36 (73.5%) recovered immediately. All but three patients had recovered normal phrenic motility at time of study exit. Other complications occurred in less than 1% of the patients and are summarized in Table 5. Notably, 8 (0.45%) patients developed cardiac tamponade, 16 (0.97%) vascular damage (1 arteriovenous fistula requiring surgery), 12 (0.68%) transient ST elevation events, and 9 (2 intraprocedural and 7 in the 30-days post ablation) patients had a cerebral stroke or transitory ischemic attack (TIA). In total, major adverse cardiovascular effects (MACE; including stroke/TIA, ST-segment elevation, and cardiac tamponade) occurred in 29 (1.6%) patients. No intraprocedural deaths occurred.

**Table 5:** Procedural related complications.

Complication	n (%)^a^
Total	120 (6.9)
**Acute complication (during procedure)**	
Phrenic nerve palsy	49 (2.30)
Resolved by discharge	36 (2.21)
Resolved by study exit	10 (0.61)
Unresolved at 12 months	3 (0.18)
Cerebral stroke/TIA	2 (0.1)
Cardiac tamponade	8 (0.45)
Transient ST elevation	12 (0.68)
Hemoptysis	1 (0.05)
Major bleeding	2 (0.11)
Other	3 (0.22)
**Subacute complication (up to 30 days post-procedure)**	
Peripheral vascular damage	17 (0.97)
Cerebral stroke/TIA	7 (0.40)
Atrio-esophageal fistula	1 (0.05)
Clinical gastroparesis	1 (0.05)
Other	17 (0.97)

*TIA* transient ischemic attack.

^a^Percentage related to the entire population.

Five deaths (0.29%) occurred during follow-up, 2 (0.12%) of which occurred during the first 30 days after the procedure. One death within 30 days was due to a traumatic cerebral hemorrhage and the other due to a mesenteric embolism (the patient was not taking the correct anticoagulant dosage). Three patients died between 140 and 308 days post procedure, one patient died as a result of lung neoplasia, one due to a complication after a left atrial appendage occlusion, and one due to an asystole documented bay emergency service (no more data were collected). In 1 patient an atrio-esophageal fistula occurred during the first month after ablation. The patient presented with global sepsis. This complication was resolved surgically, but the patient suffered serious neurologic damage. No clinical PV stenoses were reported during follow-up. At the end of the study, 95.8% of the adverse events were resolved.

### Follow-up and AF recurrence predictors

**Figure 3:**
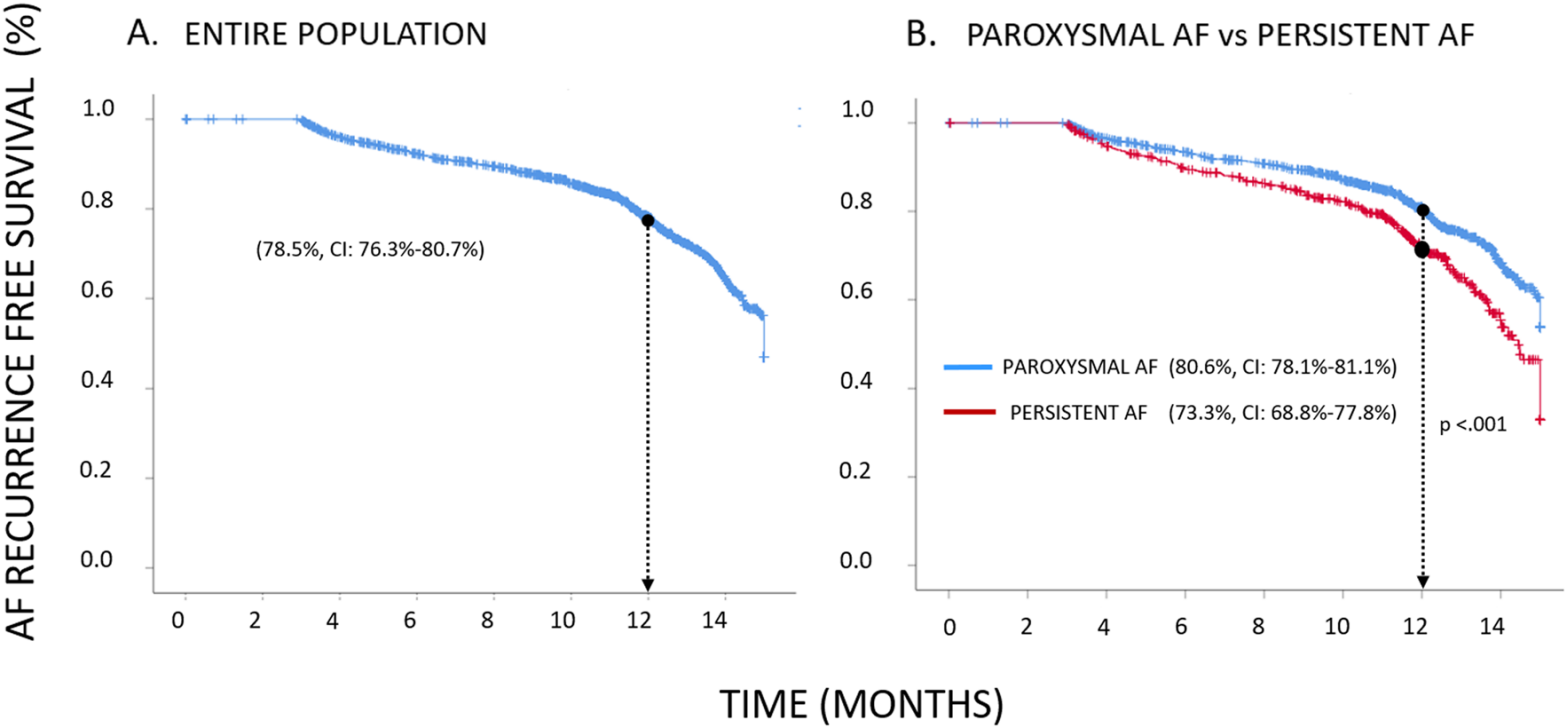
Kaplan–Meier survival curves for AF recurrence at 12-months for all patients (**A**). Comparison between PAF versus perAF (**B**).

Of the 1742 patients enrolled in the RECABA, 1628 (93.4%) completed 12-month follow-up (6.54% lost to follow-up rate, mostly due to restrictions caused by the Covid-19 pandemic) with a median follow-up of 375 (IQR 342–415) days since the index procedure. Arrhythmic event monitoring was conducted with electrocardiography (48.6%), 24-h Holter monitoring (39.9%), 72-h Holter monitoring (3.6%), implantable continuous loop recorder (2%) and non-implantable continuous recorders (3.4%). The 12-month Kaplan–Meier estimate of freedom from AF recurrence after the blanking period was 78.5% (95% CI 76.3–80.7%; Fig. 3A). The 12-month estimate of freedom from a ≥ 30 s recurrence of AF in patients with PAF was superior to those with PerAF (80.6% CI 78.1–81.1% vs 73.3% CI 68.8–77.8% respectively; p < 0.001; Fig. 3B).

**Figure 4:**
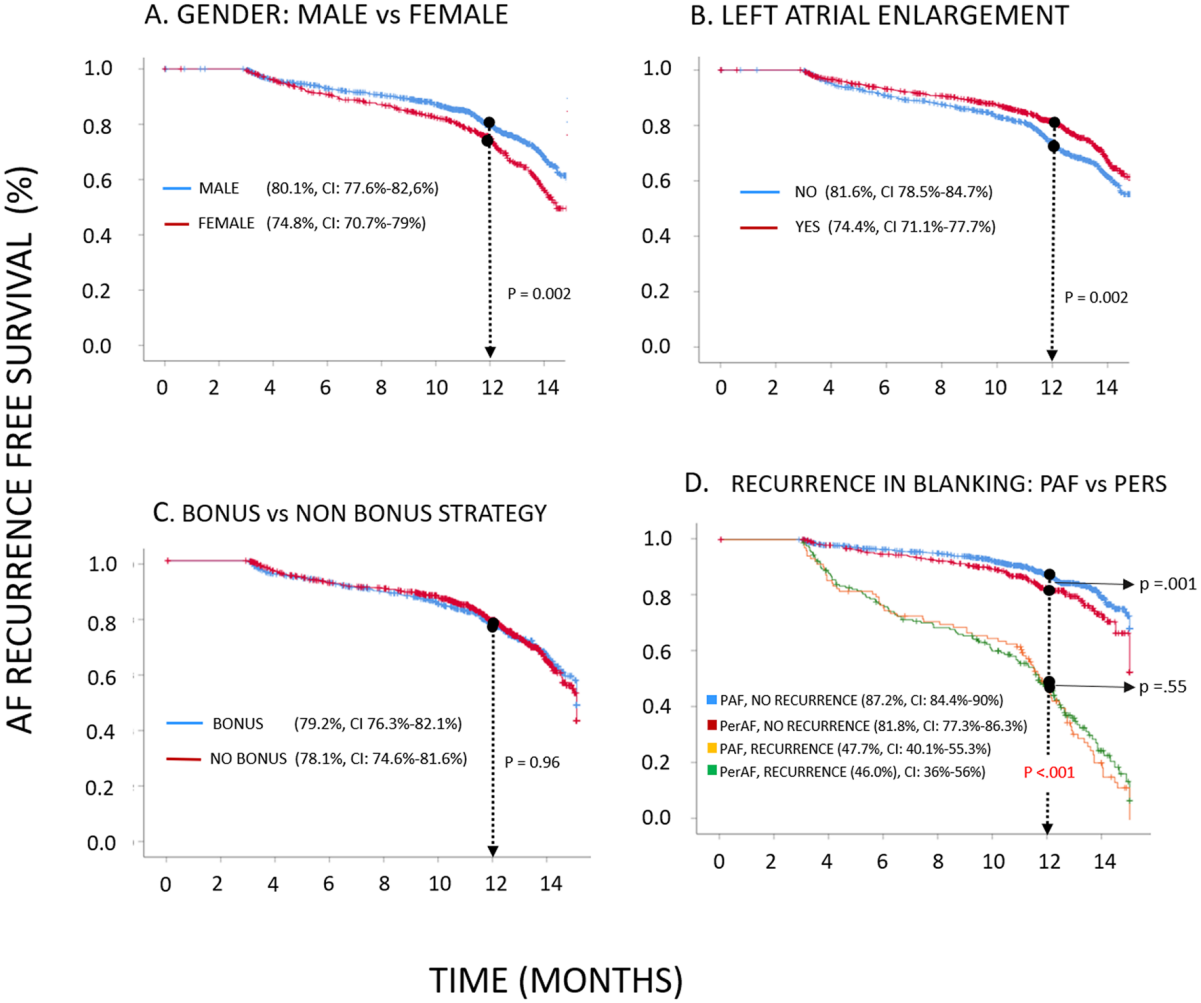
Kaplan–Meier survival curves for AF recurrence at 12-months. Analysis of different predictive factors for AF recurrence.

Baseline patient characteristics and procedural variables were analyzed to identify predictors of AF recurrence (Table 6). Univariate analyses identified the following baseline characteristics predicted AF recurrence: ≥ 65 years of age, female gender, non-PAF, CHA_2_DS_2_-VASc ≥ 2, no physical activity, structural heart disease, LVEF ≤ 50%, and LA enlargement. The following predictors were found close to the limit of significance: using a bonus strategy (p = 0.098; 95% CI 0.96–1.55) and duration of AF history > 1 year (p = 0.098; 95% CI 0.95–1.83). A multivariate model identified the following independent predictors of AF recurrence: non-PAF (OR = 1.70, p < 0.001, 95% CI 1.31–2.20), LA enlargement (OR = 1.35, p = 0.017, 95% CI 1.05–1.73), and female gender (OR = 1.33, p = 0.039, 95% CI 1.01–1.76). In total, 276 patients (17%) had AF recurrence during the blanking period, and AF recurrence during the blanking period was a significant post-procedural predictor of arrhythmia recurrence (HR = 5.98, p < 0.001, 95% CI 4.96–7.20; HR = 6.06, p < 0.001, 95% CI 4.93–7.46 for univariate and multivariate analysis respectively). Figure 4 shows Kaplan–Meier free survival curves for highlighted independent and potential predictors AF recurrence.

**Table 6:** Predictors of AF recurrences.

	Univariate			Multivariate		
	OR	OR 95% CI	p	OR	OR 95% CI	p
**Clinical parameters**						
Age (≥ 65 years)	1.46	1.16–1.85	0.002	1.15	0.87–1.51	0.324
Gender (female)	1.41	1.12–1.76	0.003	1.33	1.01–1.76	0.039
Persistent AF	1.77	1.40–2.22	< 0.001	1.70	1.31–2.20	< 0.001
Time since diagnosis (> 1 year)	1.32	0.95–1.83	0.098	1.38	0.98–1.96	0.068
CHA2DS2-VASc (≥ 2)	1.55	1.25–1.93	< 0.001	1.18	0.89–1.55	0.252
No physical exercise	1.35	1.09–1.68	0.007	1.10	0.86–1.39	0.449
Structural heart disease	1.36	1.04–1.77	0.024	1.11	0.78–1.56	0.563
Left ejection fraction (≤ 50%)	1.42	1.00–2.01	0.050	1.04	0.64–1.70	0.866
Left atrium enlargement	1.59	1.27–1.99	< 0.001	1.35	1.05–1.73	0.017
Heart failure	1.41	0.97–2.05	0.070	0.87	0.53–1.46	0.590
**Procedure parameters**						
Bonus strategy	1.22	0.96–1.55	0.098	0.87	0.68–1.13	0.308

	HR	HR 95% CI	p	HR	HR 95% CI	p
**Post-procedure parameters**						
Early AF recurrence	5.98	4.96–7.20	< 0.001	6.06	4.93–7.46	< 0.001

**Figure 5:**
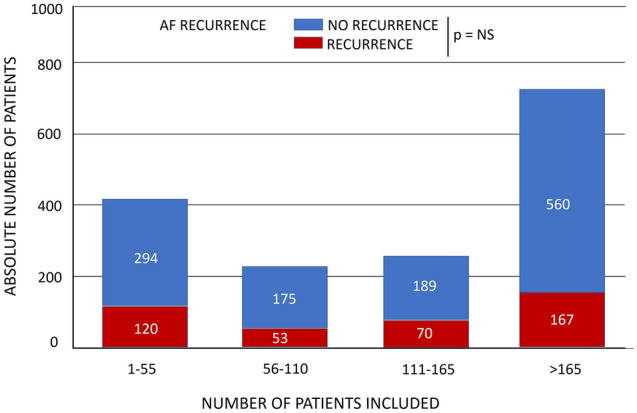
AF recurrence and no-recurrence at 12-months of follow-up (absolute number of patients) by center experience. Centers were divided into four quartiles of expertise with a fixed range of 55 patients in each quartile, increasing in the level of experience. There was not statistical difference regarding either overall recurrence at 12-month follow-up or the rate of adverse events. Procedure characteristics were different between quartiles (Table S2).

The AF recurrence rate did not differ based on center volume in this study (p = ns) (Fig. 5). However, procedure time, fluoroscopy time, cryoablation time, and percentage of isolated veins was improved in more experienced centers, and a bonus cryoapplication strategy was used more often in experienced centers (all p < 0.001). The adverse event rate was not different between centers grouped by experience (p = 0.1) (“S4”, Table S2, “Supplementary Material Online”).

### Medications during follow-up

After CBA, 89.8% of patients were discharged after 1 day or less (median time to discharge 1 day; mean 1.25 ± 2.01 days). Although there were no protocol requirements for AAD usage, medication data were collected. At time of discharge, 1131 patients (65%) were taking AAD, most frequently Class Ic agents (690 patients; 61.2%) and amiodarone (333 patients; 29.5%). All patients were taking an OAC agent at discharge (1270; 72.9% patients taking direct OAC).

**Figure 6:**
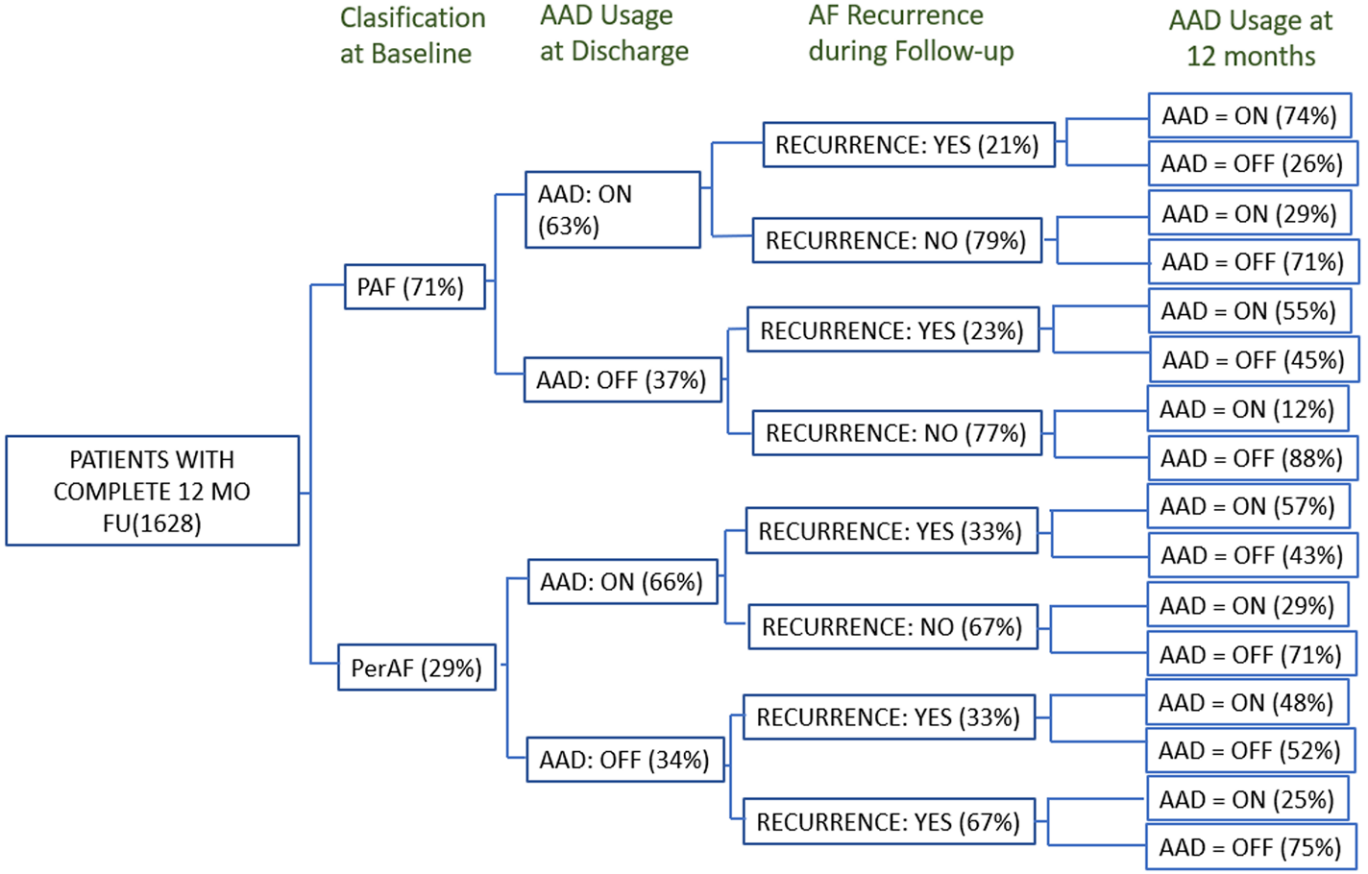
Flow-chart showing AF recurrence data according to type of AF, AAD at discharge and AAD usage at the final follow-up for the entire population. (see text for details).

Of the 1628 patients who completed follow-up, 1038 were on AADs (63.8%), with a similar proportion in PAF (63%) and PerAF (65.8%). The percentage of patients free of AF recurrence on AADs at 12-month follow-up was also similar between PAF and PerAF groups (29.5% vs 28.9 respectively, p = ns). Patients discharged under AAD treatment were more likely to continue AADs at the end of follow-up, irrespective of AF recurrence (29% versus 12% in the PAF group; p = 0.002). Figure 6 displays the cohort according to type of AF, AAD at discharge, recurrences and AAD at the final follow-up.

## Discussion

The RECABA registry is the largest cryoballoon registry in Spain and reflects the standard clinical practice and clinical characteristics of patients treated by CBA for PVI in Spanish hospitals. AF is the most frequently ablated arrhythmia in Spain (27.8% of all arrhythmias), and last year, 42% of these procedures were performed with cryoballoon^9^. The results of RECABA demonstrated acute and subacute complications associated with real-world usage of CBA for treatment of AF were rare (6.9%), and cryoablation was effective with 79% freedom from AF at 12-month when used according to standard clinical practice in 27 unique centers in Spain.

### Tailored and efficient cryoballoon ablation procedure

Patients in RECABA were mostly young, overweight adult males who presented with PAF without structural heart disease and are consistent with baseline characteristics of patients enrolled in other European, multicenter registries^7,8,14^. The cryoablation procedure was frequently performed under superficial sedation, which has been reported to result in shorter procedure times without compromising AF recurrences or complication rates^15^. During recent years, cryothermal dosing protocols have also evolved towards shorter application times based on per-PV TTE with or without bonus applications, and have resulted in more efficient procedure times than fixed cryoapplication times^16–18^. This trend was also observed in RECABA in which two thirds of the centers systematically used dosing protocols without bonus applications that led to significantly fewer applications per patient and shorter procedure, cryotherapy, and LA times. Centers with more experience applied the bonus strategy to a greater extent; although, there is no clear explanation for this observation. It is possible these centers perceived a greater need for applications as they treated more patients with PerAF; thus, it is important to attempt PV potential monitoring during ablation to tailor dosing to each PV. In the RECABA registry, PV potentials could be seen during ablation in 61% of the veins and more often in left PVs. These results are similar to contemporary reports, and rates have improved with the introduction of new generations of cryoballoon^16–19^. General procedure times in the RECABA registry, such as cryotherapy, LA and x-ray exposure times were comparable to those obtained in other studies, and they were significantly reduced in non-bonus protocols^7,9,14,16^. These data corroborate a trend toward simplifying and maximizing efficiency of AF ablation procedures.

### Antiarrhythmic drugs on long-term outcomes

There are mixed reports on the influence of short-term use of AADs on long-term outcomes after AF ablation^20–22^. The European survey of AF ablation practice that included 1300 patients from 72 European institutions found that among a patient population that was more than two-thirds PAF, 65% of patients were discharged post-ablation on an antiarrhythmic medication and 49% remained on antiarrhythmic medication at 12 months^23^. The RECABA study observed similar rates of AAD usage after discharge. Although further randomized data are required, patients discharged on AADs were more likely to continue AAD use throughout study follow-up even in the absence of arrhythmia recurrence. The reasons for this may range from a perception of added efficacy of the AAD or its additional effect on the ablation, but more probably it is due to the nature of a registry and lack of strict control in the follow-up. In the group of PerAF, however, the differences were non-significant (Fig. 6).

### Predictors of outcomes after cryoablation

Independent predictors for AF recurrence were female sex, non-PAF, LA enlargement, and early recurrence in blanking period. Non-PAF and LA enlargement are a well-established predictors of AF recurrence^24^. In the recent CRYO4PERSISTENT AF Trial^25^, a controlled multicenter study, a single CBA for treatment of PerAF demonstrated 61% success at 12 months and improved quality of life which is in accordance with the data of the current study. A sub-analysis of the Fire and Ice Trial showed that female sex was associated with an almost 40% increase in the risk of AF recurrence and cardiovascular rehospitalization after PV isolation^26^. In the RECABA study we found early recurrence in blanking period increased the risk for late AF recurrence sixfold. Lack of recurrence in the blanking period, however, had less predictive power in the PerAF group. In accordance with this finding, early AF recurrence in blanking period (classically considered as non-valuable and unrelated with late AF recurrence, especially in radiofrequency PV ablations) has been found as a strong predictor of long-term AF recurrence in several recent studies^27^, especially when it occurs in the second or third month after ablation. These data align with some authors suggestion that recurrences during the blanking period should be redefined^28^.

Interestingly, the independent predictors of AF recurrence within the RECABA registry were in general alignment with the findings of large-randomized trials, as well as the safety and efficacy data. Consequently, the RECABA results demonstrate the value of using real-world registries to further validate study findings from randomized controlled trials.

## Study limitations

The RECABA registry was a multicenter prospective observational registry; therefore, acknowledged limitations include potential bias in patient selection, varied patient management, and the lack of a control group. Nevertheless, possible biases are mitigated by the fact that data were collected prospectively, and research endpoints were pre-specified. As per nature of this project, a standardized-cryoballoon dosing protocol was not implemented. Few patients were implanted with an internal loop-recorder; therefore, asymptomatic episodes may have occurred unnoticed, and our success rate may have been over-estimated. Complications that occurred during the ablation procedure may be registered adequately in a registry, but the voluntary nature of data provision may have led to underreporting of procedural complications after the ablation. No recommendations were provided to the participating centers in terms of pharmacological treatment following CBA. Thus, the exact temporal sequence of AAD management could not be established, and it is unknown whether AADs were simply continued after the blanking period as per center practice. Multivariate predictive models could be affected by variables not considered in the particular model (residual confounding), since only statistically significant predictors have been considered. Being an observational study, no effort has been made to control for covariates by sampling design.

Despite limitations, this prospective research may provide a representative analysis of the real-world outcomes of CBA in a broad cohort of patients with AF in a large number of centers with different levels of experience.

## Conclusions

Cryoablation was effective and safe when used according to standard clinical practice during the early cryoballoon ablation experience at 27 unique centers in Spain. LA enlargement, non-PAF, female sex and early AF recurrence in the first 3 months independently predicted late AF recurrence.

### Perspectives

#### Clinical competencies

- Cryoablation for the treatment of AF performed according to standard clinical practice in 27 centers was safe and effective.
- Cryoballoon ablation is commonly delivered with a tailored, efficient approach.
- Female sex, non-PAF, LA enlargement, and early recurrence in blanking period predicted recurrence during 12-month follow-up.

#### Translational outlook

Cryoballoon ablation is used to treat a broad group of patients with atrial fibrillation and results in consistent outcomes.

## Supplementary Information

Supplementary Information.

## Data Availability

The data that support the findings of this study are available from the corresponding author, A.F., with permission of the Medtronic team upon reasonable request.

## Abbreviations

AF: Atrial fibrillation
PV: Pulmonary vein
PVI: Pulmonary vein isolation
AAD: Antiarrhythmic drugs
CBA: Cryoballoon ablation
RFA: Radiofrequency ablation
PAF: Paroxysmal atrial fibrillation
PerAF: Persistent atrial fibrillation
OAC: Oral anticoagulation agents
TIA: Transient ischemic attack
MACE: Major adverse cardiovascular event
