# Supplementary material for: Primary results of the Spanish Cryoballoon Ablation Registry: acute and long-term outcomes of the RECABA study
Source: Sci Rep. 2021 Aug 26;11:17268. doi: 10.1038/s41598-021-96655-3 (PMC8390492; doi:10.1038/s41598-021-96655-3)
Supplement: Supplementary file 1 — Supplementary Information. [file 41598_2021_96655_MOESM1_ESM.docx]

**SUPPLEMENT APPENDIX**

**Manuscript Title:** Acute and Long-term Outcomes of Cryoballoon Ablation in Patients with Atrial Fibrillation. Primary Results of the Spanish Cryoballoon Ablation Registry (RECABA)

**Brief Title:** Results of the Spanish Cryoballoon Ablation Registry (RECABA)

**ClinicalTrials.gov Identifier:** NCT02785991

**Contents:**

- Table S1: Spanish centers and collaborators participating in RECABA

- S2: Data collection, management, and quality control.

- S3: Description of cryoballoon ablation procedure.

- S4: Description of analyses by center experience.

- Table S2. Analysis of general characteristics by center experience.

**Table S1. Spanish Centers and collaborators participating in RECABA.**

| ***Nº*** | ***Center*** | ***n*** | ***Collaborator*** |
| --- | --- | --- | --- |
| 1 | Hospital Virgen de la Victoria, Málaga | 204 | Alberto Barrera Cordero |
| 2 | Hospital Puerta de Hierro, Madrid | 188 | Jorge Toquero Ramos |
| 3 | Hospital Basurto, Bilbao | 145 | Jesús Daniel Martínez Alday |
| 4 | Hospital Virgen de la Arrixaca, Murcia | 121 | Arcadio García Alberola |
| 5 | Hospital Clínico Universitario, Valencia | 120 | Ángel Ferrero de Loma-Osorio |
| 6 | Hospital Virgen Macarena, Sevilla | 100 | Rocío Cózar León |
| 7 | Fundación Jiménez Diaz, Madrid | 98 | José Manuel Rubio Campal |
| 8 | Hospital del Mar, Barcelona | 96 | Ermengol Vallés Gras |
| 9 | Clínica Zorrotzuarre, Bilbao | 74 | Jose Miguel Ormaetxe Merodio |
| 10 | Hospital Quirón, Valencia | 58 | Ricardo Ruiz Granell |
| 11 | Hospital Trias i Pujol, Barcelona | 56 | Roger Villuendas Sabaté |
| 12 | Hospital Vithas, Sevilla | 53 | Pablo Bastos Amador |
| 13 | Hospital La Paz, Madrid | 51 | Rafael Peinado Peinado |
| 14 | Complejo Hospitalario Universitario, A Coruña | 50 | Luisa Pérez Álvarez |
| 15 | Hospital San Juan, Alicante | 43 | Jesús Castillo Ballesteros |
| 16 | HU de Canarias, Santa Cruz de Tenerife | 42 | Aníbal Rodríguez González |
| 17 | Hospital Juan Ramón Jiménez, Huelva | 40 | Pablo Moriña Vázquez |
| 18 | Hospital Virgen de las Nieves, Granada | 37 | Luis Tercedor Sánchez |
| 19 | H Ntra Señora de la Candelaria. Tenerife | 32 | Julio Hernández Alfonso |
| 20 | Hospital de Araba, Vitoria | 31 | Mª Fe Arcocha Torres |
| 21 | Hospital Son Espases, Mallorca | 28 | Carlos Grande Morales |
| 22 | Hospital La Fe, Valencia | 18 | Oscar Cano Pérez |
| 23 | Hospital Josep Trueta, Gerona | 15 | Emilce Trucco Vernetti |
| 24 | Hospital Reina Sofía, Córdoba | 14 | Jose Mª Segura Saint-Gerons |
| 25 | Hospital Virgen de la Salud, Toledo | 14 | Cristina Martín Sierra |
| 26 | Hospital Rey Juan Carlos, Madrid | 10 | Elena Mejía Martínez |
| 27 | Hospital General Universitario, Alicante | 4 | Alicia Ibáñez Críado |

**S2: Data collection, management, and quality control**.

Clinical data were collected at the baseline procedure and at the annual follow-up by the coordinator responsible for each center through an electronic data collection system for clinical trials (eCRF). The data from the CRF was stored in a secure, password-protected data base. Personal data were always processed with due confidentiality and privacy and in accordance with the applicable regulations on data protection and privacy. There were no additional tests, and only the necessary data were collected in accordance with the variables considered in the Case Report Form. The study was conducted in compliance with the most recent version of the Declaration of Helsinki.

The main data collected included: 1) Baseline data: demographic factors, cardiovascular risk factors (arterial hypertension, diabetes mellitus, dyslipidemia, smoking), physical activity, sleep apnea, body mass index, evolution time of the arrhythmia, previous anticoagulant and anti-arrhythmic treatments, previous cardioversions, anatomical data arising from the imaging tests (left ventricular parameters, ejection fraction, ventricular wall thickness, diameter, area or volume of the left atrium (LA), pulmonary vein drainage pattern). 2) Procedure variables included: number of applications per vein, application time, registration of PV potentials during the ablation, time to block the vein (TTE), minimum temperature reached, reheating time, isolation or not of the PV, procedure time and x-ray exposure times, temperature details in each of the applications, temperature at the time of PVI and acute complications. 3) Annual follow-up variables included: anti-arrhythmic treatment, clinical recurrence of arrhythmia and follow-up method according to the protocol for each site.

**S3: Description of cryoballoon ablation procedure.**

By transeptal approach, a deflectable 15 F sheath (FlexCath Advance^®^; Medtronic CryoCath, Kirkland, Quebec, Canada) was advanced toward the LA. For ablation, a 10.5 F cryoballoon catheter, 23 or 28 mm in diameter (Arctic Front Advance^®^, Medtronic, Inc., Minneapolis, MN, USA), was introduced through the deflectable sheath. Pacing and electrical activity recording from inside the pulmonary vein (PV) was performed with a 3.3 F octapolar mapping electrode catheter either 15 or 20 mm in diameter (Achieve™; Medtronic, Inc.). Once a PV was catheterized, the cryoballoon was inflated and advanced toward the PV ostium. PV angiography was done or not depending on the protocol of each center. The electrode catheter was then shifted proximally to record potentials inside the vein. When PV potentials could be recorded during application, time to effect (TTE) was defined as the time from freezing initiation to vein potential entrance block. An entrance block was defined as the absence of a detectable PV potential inside the PV. An exit block (in sinus rhythm) was defined as the detection of a local capture inside the vein during pacing with the electrode catheter, with no evidence of atrial capture; or a recording of spontaneous activity inside the PV, dissociated from the LA. When an optimal occlusion of the PV was not achieved and a PV was not isolated during the application, maneuvers reported in the literature to increase contact between the cryoballoon and the venous ostium (pull-down or hockey-stick maneuvers) were performed. Alternatively, the octapolar mapping catheter was repositioned in different branches or more deeply inside the PV to optimize the cryoballoon orientation and/or stability. Cryo-dosing protocol in terms of duration of applications or bonus/non-bonus-freeze strategy, post-ablation waiting time, or adenosine test was different according to the usual practice of each hospital. During right PV applications, the phrenic nerve was stimulated through the superior vena cava to detect potential phrenic nerve injury during cryoablation. When phrenic nerve injury was observed, the application was halted immediately with the double-stop technique. Esophageal temperature was not measured routinely during the procedure. The acute procedure endpoint was defined as a persistent, bidirectional PV block.

**S4: Description of analyses by center experience**

Centers were divided into four quartiles of expertise with a fixed range of 55 patients in each quartile, from level one (less experience) to level 4 (more experience). There was not a significant statistical difference regarding overall recurrence at one-year follow-up and adverse events. However, we found significant differences regarding to procedure characteristics that are shown in Table S2.

**Table S2. Analysis of general characteristics by center experience.**

| ***Center Level*** | ***LEVEL 1*** | ***LEVEL 2*** | ***LEVEL 3*** | ***LEVEL 4*** | ***p*** |
| --- | --- | --- | --- | --- | --- |
| ***Number of patients*** | 1-55 | 56-110 | 111-164 | ≥ 165 |  |
| ***Patients with PAF (%)*** | 72.5 | 78.1 | 77.6 | 66.6 | <.001* |
| ***Patients with PersAF (%)*** | 27.5 | 21.9 | 22.4 | 33.4 | <.001* |
| ***Procedure time (min)*** | 130±51 | 118±35 | 120±40 | 99±31 | <.001 ^ϯ^ |
| ***Rx time (min)*** | 29±19 | 29±14 | 27±14 | 18±13 | <.001 ^ϯ^ |
| ***Total cryothermia time (min)*** | 22±8 | 18±7 | 21±7 | 21±7 | <.001 ^‡^ |
| ***PVI (%)*** | 89±2 | 88±2 | 97±1 | 96±1 | <.001^ꬸ^ |
| ***Bonus Strategy (%)*** | 19.1 | 0.9 | 53.7 | 38.7 | <.001^ꬸ^ |
| ***Adverse Events at 30 days (%)*** | 8.5 | 5.3 | 3.9 | 6.5 | 0.102 |

* for all centers; ϯ difference between level 4 centers and the rest; ‡difference between level 2 centers and the rest; ꬸ difference between level 3 and 4 centers and the rest.
